# Supplementary material for: Effect of the alveolar recruitment maneuver during laparoscopic colorectal surgery on postoperative pulmonary complications: A randomized controlled trial
Source: PLoS One. 2024 May 9;19(5):e0302884. doi: 10.1371/journal.pone.0302884 (PMC11081303; doi:10.1371/journal.pone.0302884)
Supplement: Attachment — Submitted filename: Response to Reviewers.docx [file pone.0302884.s003.docx]

At manuscript

⇒ Thank you, Line 291 changed, “The author(s) received no specific funding for this work.”

Reviewer #3: Thank you for responding well to suggestion provided during previous review.

Only several minor revision:

1. in Line 40, i think there is no "dot" after the standard deviation and should be a continuous sentence. please confirm

⇒ Thank you for pointing out the error. The “dot” was deleted and modified to one sentence.

2. Please add in abstract in line 45 that the RM did not had any significant side effect (addresing "safety" of maneuvre using short few words") : "undergoing laparoscopic colorectal surgery and was not associated with any significant adverse events"

⇒ We added the sentence as your recommendation.

3. in line 218: please simplify this sentence: suggestion: "The importance of preventing PPCs must be emphasized, given that.." (the use of cannot be overemphasized is confusing for certain english reader, as this sentence followed by emphasizing the effect of prolonged LOS and expenses)

⇒ The sentence is corrected as your recommendation.

3. in line 222, please add driving pressure findings as well as it has been presented in result section. can be just simply: "...significantly reduce PIP and DP...."

⇒ We corrected the sentence as your recommendation.

4. please add in the safety aspect of RM performed with short few words in line 281 after the "calculated compliance": suggestion: "......increased the calculated compliance, without posing any significant adverse events during the intervention"

⇒ We corrected the sentence as your recommendation.

Thank you for these constructive advices for this paper.

Reviewer #4: Thank you for addressing the comments. I am satisfied with the revisions made.

The authors have addressed all the relevant issues that were highlighted.

Reviewer #5: (No Response)
