# Supplementary material for: Effect of the alveolar recruitment maneuver during laparoscopic colorectal surgery on postoperative pulmonary complications: A randomized controlled trial
Source: PLoS One. 2024 May 9;19(5):e0302884. doi: 10.1371/journal.pone.0302884 (PMC11081303; doi:10.1371/journal.pone.0302884)
Supplement: Attachment — Submitted filename: Reviewer comments.docx [file pone.0302884.s002.docx]

Answer to the Review Comments

**Reviewer #1: Thanks for letting me review the manuscript entitled " the effect of alveolar recruitment maneuver during laparoscopic colorectal surgery on postoperative pulmonary complications: A randomized controlled trial".
In this study, the authors intend to explore the role of alveolar recruitment maneuver during intraoperative mechanical ventilation, which is a meaningful topic. However, this study has some major defects, such as unreasonable design, unclear key contents, unclear language and logic, so it is not recommended to accept it.
The main shortcomings are as follows:
(1) In low-risk patients undergoing laparoscopic colorectal cancer resection, the incidence of postoperative pulmonary complications is about 30% in this study, which is not in accordance with the clinical practice and is obviously contradictory to the previous studies. This is a design defect and cannot be improved by later modification.
(2) The treatment is not clear described, and there are no references.
(3) The definition of the primary outcome is unclear, and the definition of postoperative pulmonary complications is not clear and unreasonable.
(4) The statistics are obviously insufficient, missing too much cases, not using intention-to-treat analysis, etc.
(5) The authors did not quote the literatures correctly.**

**Reviewer #2: Overall this was a simple and well conducted trial. There are 3 areas of concern that need to be addressed

1. Clearly, the adequacy of the recruitment manoeuvre (RM) chosen is important to the success of the trial. The authors have chosen a low-level static RM with a Paw of 30 cm H2O for 30 sec but with no justification of this level and with no literature citing that this should be an effective RM in this clinical setting.
In non-operative patients with ARDS, RMs range from stepwise dynamic RMs with PEEP up to 40 cmH2O with tidal PCV up to 55cmH2O for 2 min, to static RMs with Paw 40 cmH2O for 40 sec. The RM in this study is well below this range.
The authors have cited 2 papers that included peri-operative RMs but with no description of the level of RM used to support the use of the RM chosen for use in this study.
Thus it is possible that the RM used was simply not high enough and/or not supported by sufficient PEEP.
The Conclusion in the Abstract and at the end of the paper states “The alveolar recruitment maneuver did not further reduce postoperative pulmonary complications”
This implies that any RMs may not work on this setting.
The Conclusions should specify the level of RM used so it is clear that the results apply to this RM.
“The alveolar recruitment maneuver with Paw 30 cmH20 for 30 sec and PEEP 5 did not further reduce postoperative pulmonary complications”
This should also receive discussion in the section of the paper devoted to study limitations**

⇒ We specified the method of RM (including pressure) at the Abstract (page 2 line 35, page 2 line 43) and Conclusion(page 16 line 277).

We corrected the Discussion to justify our selection of the RM specifically describing the pressure used in the references (page 14 line 230-48).

We added the Paw 30 cmH2O we chose as the limitation of this study (page 15 line 259-261).

**2. The reduction in PIP in the Recruitment Group has been attributed to improved lung compliance following the RM. This may have been a beneficial effect of the RM, but it may also have been the use of lower tidal volumes in this unblinded study. Clearly the tidal volumes were measured so that they could be set in the range of 6-8ml/kg for recording of the PIPs presented in the paper.
It is important to the results also record the tidal volume and calculated lung compliance at each measurement time before any conclusions in relation to the effects on PIP can be made.**

⇒ We added the calculated lung compliance at each time point as Figure 2. (page 12 Fig 2). In Fig 2, we used driving pressure instead of PIP as the recommendation of Reviewer#3.

**3. The table in relation to PPCs and the overall frequency of PPCs is ambiguous and need clarification. The paper reports a PPC rate of 30%.
It is possible that each complication occurred in a separate patient resulting in 30% of patients having a PCC, OR it is possible that all PCCs occurred in the 17-20% of patients with atelectasis resulting in 18% of patients having a PCC.
The abstract, table and discussion should all also quote the total % of patients who developed a PCC**

⇒ We clarified the definition of PPC at the Method (page 6 line 120-2) and Table 2(page 10 line 180-3)

**Reviewer#3: Thank you authors for succesfully completed this research. This research is important to evaluate the efficacy of important intervention which is a pragmatic practice usually performed intraoperatively in the attempt to evaluate this strategy to further reduce post-operative pulmonary complication (PPC) in patients at high risk for PPC.

Overall, authors wrote a comprehensive, detailed explanation regarding the importance of this study, clear research question and methodology which is reproducible by other Anaesthesiologist and clear intention and primary outcome investigated for.

However there are two major issues with regard to the primary outcome:

1. Primary outcome is not clear
--> Primary outcome of PPC is not clear as a distinct composite outcome as author presented PPC in Table 3 as separation of PPC into symptoms, signs and clinical feature of PPC rather than PPC stand-alone as a composite outcome as usually being reported by other research in the field of PPC.
-- For example: one patient could have Hypoxemia in PACU, as well as Pleural Effusion in the ward later on, thus the question will arise as whether this patient considered as 1 PPC or 2 PPC? as per convention and usual practice, PPC in this case is considered only 1 incidence despite having 2 clincal signs at two different time in the same patient.
-- Further explanation regarding PPC as a composite outcome rather than separate distinct clinical signs can be considered here in** [**https://doi.org/10.1093/bja/aex002**](https://doi.org/10.1093/bja/aex002) **and example of composite outcome of PPC reported can be found here in Table 3 in this study here** [**https://doi.org/10.1371/journal.pone.0274749**](https://doi.org/10.1371/journal.pone.0274749)

⇒ We clarified the definition of PPC at the Method (page 6 line 120-2) and Table 2(page 10 line 180-3)

**2. Please include line chart indicating the changes of pressure over time with regarding to Peak inspiratory pressure in both group and compare from initial time at induction, gas-in, gas-out and recovery changes and compare with proper statistical anlysis, such as Repeated Measures ANOVA (RMANOVA) to study the significance changes drop over time for within the group and comparative statistical changes between the group over the time course using RMANOVA. this test is more appropriate than student t-test used for table 2**

⇒ We added the trends of driving pressure and calculated compliance as Figure 2 instead of Table 2 (page 12). ANOVA was used for statistics (page 8 line 150-1, page 12 Figure 2). In Fig 2, we used driving pressure instead of PIP as the recommendation of Reviewer#3**.**

**There are some other minor issues such as:

1. Table 1 presented baseline data without statistical analysis comparison between the two group to provide the readers to study and ensure both groups have comparable baseline characteristic, thus permitting comparison of intended primary outcome as the baseline was similar in both group**

⇒ We added statistics to Table 1 (page 9).

**2. Safety assessment of intervention of alveolar recruitment in this study need to be presented as this was planned based on the research proposal provided: "If low blood pressure occurs during the study, general treatment such as intravenous fluid administration and administration of vasopressors will be administered at the discretion of the anesthesiologist in charge. If serious complications occur due to ventilation therapy after surgery, immediately report them to the Seoul National University Bundang IRB in writing via phone or email"
--> please present any incidence of BP low or require IV fluid or vasopressor during or due to alveolar recruitment intervention**

⇒ We added the data of intraoperative hypotension and the need for vasopressor at Results (page 12 line 198~page 13 line 202).

**3. Allocation ratio into groups intervention and control was not mentioned, but it was implied to be 1:1. please mention in the text**

⇒ We mentioned it at the Method (page 5, line 88).

**4. In addition to or instead of Peak Inspiratory Pressure, please consider including driving pressure calculated by (Peak Insp minus PEEP) as part of secondary outcome as many research and convention and consensus now studying effect of driving pressure to multiple various outcome in mechanical ventilation**

⇒ We showed the trend of driving pressure and calculated compliance at Figure 2 (page 12).

**5. Primary outcome table should come before secondary outcome table. Table 2 and 3 should switch the order**

⇒ We switched the order of Table 2 & 3 and Table 3 was replaced with Figure 2 (page 10-12).

**Reviewer #4: Nice attempt to explore the intervention. Methodologically sound but not novel to this field and provides no new insight or information on this topic. I commend the effort but this study does not add any value to the wealth of of knowledge on this topic. The design of the study should be re-looked and the PEEP strategy needs refinement to meet better end-points i.e. PEEP titration to compliance, blood gases or EIT
I feel this study is not clinically adding any insights to the field and should be re-examined for relevance. It would be more relevant if some other tool such as electrical impedance tomography(EIT) determined what the optimal PEEP should be for the individual patients.**

**Reviewer #5: Manuscript PONE-D-23-24336 is a randomized controlled trial on perioperative alveolar recruitment and its effect on postoperative pulmonary complications. The study is well performed and written. Analysis is sound and data support the conclusions.

Comments.
1. In table 2 PIP is expressed in cmH2O, in the legend mmHg is indicated.**

⇒ We corrected the legend to cmH2O (page 12 Figure 2). Table 2 was replaced by Figure 2.

**2. Page 14, last sentence of first paragraph: 'Nevertheless, a reduction ....... risk of PCCs.' This remark is not supported by the data.**

⇒ We deleted this sentence and modified the Discussion (page 14 line 226-7).

**3. Page 15, why was a high FiO2 of 0,8 -1,0 used in anesthesia recovery? Is this protocollized? Why not titrated on saturation? I presume that, with a base fiO2 of 0,4 during anesthesia, FiO2 is perioperatively titrated. The authors should reflect on this issue.**

⇒ We looked into the protocol again and found that we did not protocolized the FIO2 at recovery. It was up to the attending anesthesiologists. Hence, we deleted this sentence.

**4. The authors should report the side effects of the recruitment maneuver in the studied patients.**

⇒ We added the side effects of the RM at the Results (page 12 line 198~page 13 line 202).

**** There has been a change in the reference**.

⇒ # 1, 19, 21, 22, 24, 25, 26 were deleted.

⇒ Add #1, 2

1.	Ferrando C, Soro M, Unzueta C, Suarez-Sipmann F, Canet J, Librero J, et al. Individualised perioperative open-lung approach versus standard protective ventilation in abdominal surgery (iPROVE): a randomised controlled trial. Lancet Respir Med. 2018;6(3):193-203.

2.	Karalapillai D, Weinberg L, Peyton P, Ellard L, Hu R, Pearce B, et al. Effect of Intraoperative Low Tidal Volume vs Conventional Tidal Volume on Postoperative Pulmonary Complications in Patients Undergoing Major Surgery: A Randomized Clinical Trial. JAMA. 2020;324(9):848-58.

⇒ The reference numbers were incremented by one.

#2 – #16 🡪 #3 - #17

⇒ The reference numbers were changed.

#23 -> #18, #20-> #19, #17 -> #20, #18- > #21

“My deepest appreciation goes to the reviewers.“
